# Abiotic carbonate dissolution traps carbon in a semiarid desert

**DOI:** 10.1038/srep23570

**Published:** 2016-03-29

**Authors:** Keyu Fa, Zhen Liu, Yuqing Zhang, Shugao Qin, Bin Wu, Jiabin Liu

**Affiliations:** 1 Yanchi Research Station, School of Soil and Water Conservation, Beijing Forestry University, Beijing 100083, P. R. China; 2 Key Laboratory of Soil and Water Conservation and Desertification Combating of the Ministry of Education, Beijing Forestry University, Beijing 100083, P. R. China; 3 College of Natural Resources and Environment, Northwest A & F University, Yangling 712100, Shaanxi, P. R. China

## Abstract

It is generally considered that desert ecosystems release CO_2_ to the atmosphere, but recent studies in drylands have shown that the soil can absorb CO_2_ abiotically. However, the mechanisms and exact location of abiotic carbon absorption remain unclear. Here, we used soil sterilization, ^13^CO_2_ addition, and detection methods to trace ^13^C in the soil of the Mu Us Desert, northern China. After ^13^CO_2_ addition, a large amount of ^13^CO_2_ was absorbed by the sterilised soil, and ^13^C was found enriched both in the soil gaseous phase and dissolved inorganic carbon (DIC). Further analysis indicated that about 79.45% of the total ^13^C absorbed by the soil was trapped in DIC, while the amount of ^13^C in the soil gaseous phase accounted for only 0.22% of the total absorbed ^13^C. However, about 20.33% of the total absorbed ^13^C remained undetected. Our results suggest that carbonate dissolution might occur predominately, and the soil liquid phase might trap the majority of abiotically absorbed carbon. It is possible that the trapped carbon in the soil liquid phase leaches into the groundwater; however, further studies are required to support this hypothesis.

Drylands (arid and semiarid desert ecosystems) cover about 41% of the global terrestrial surface^1^ and release a large amount of carbon via soil CO_2_ flux^2^,^3^. However, a net uptake of carbon is observed in some desert ecosystems, especially during nighttime, such as the Mojave Desert^4^,^5^, a playa of the Great Basin Desert^6^ and the Chihuahuan Desert^7^ in North America, the Gurbantunggut Desert^8^,^9^ and the Mu Us Desert^10^,^11^ in Asia and a dry valley in Antarctica^12^. Although the carbon absorptions in these cases are various, it may also play an important role in the terrestrial carbon cycle because of the great areal extent of drylands^10^,^13^. The ‘anomalous’ and variable soil carbon accretion suggests that the soil carbon cycle in desert ecosystems is quite complicated^14^,^15^.

Although it is known that CO_2_ absorption by the soil is induced by abiotic processes and is relevant to some ambient factors (e.g. temperature, soil water content, and air pressure)^8^,^10^,^12^,^16^,^17^, previous studies failed to reveal the underlying mechanism of the abiotically absorbed CO_2_ by the soil, which is essential to better understand CO_2_ absorption by the soil in drylands^18^. Applying the ^13^C isotope tracer method to undisturbed soil, we previously found that the majority of absorbed carbon might conserve in the soil solid phase^19^; however, we could not demonstrate the mechanism of abiotic CO_2_ absorption and sequestration because of the influence of biotic processes. Hence, further studies on the abiotically absorbed carbon is required.

It is surmised that abiotic carbonate dynamic is a major contributor of CO_2_ absorption by the soil^10^,^20^ and the involved abiotic processes can be expressed as^21^:


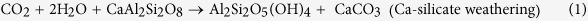



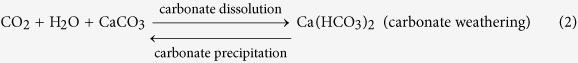


Since Ca-silicate weathering is an extremely slow process, it can be ignored at the diel or annual scale^22^, while the process of carbonate weathering may help to better explain CO_2_ absorption by the soil. However, the reactions are reversible. Whether carbonate dissolution can predominately occur and induce the abiotic atmospheric CO_2_ absorption by soil in drylands, have not been proven directly by any previous study currently. Here, we hypothesized that carbonate dissolution can induce the abiotic atmospheric CO_2_ absorption by desert soils. To test the hypothesis, we used the sterilization, leaching method and ^13^CO_2_ isotope tracer technique to investigate the abiotic soil CO_2_ exchange and ^13^C abundance of soil liquid and vapour phase in the desert soil of the Mu Us Desert, northern China.

## Results

### The amount of abiotic ^13^CO_2_ absorption

After ^12^CO_2_ was replaced in the chamber, the initial ^13^CO_2_ amount in the atmosphere of the chamber was 446 μmol. After 12 h (throughout the nighttime), the amount of the final atmospheric ^13^CO_2_ in the chamber was 149 μmol. The amount of ^13^CO_2_ absorption by the sterilised soil reached to 297 μmol.

### ^13^C abundance (δ^13^C) in the leaching solution with and without ^13^CO_2_ addition

**Figure 1:**
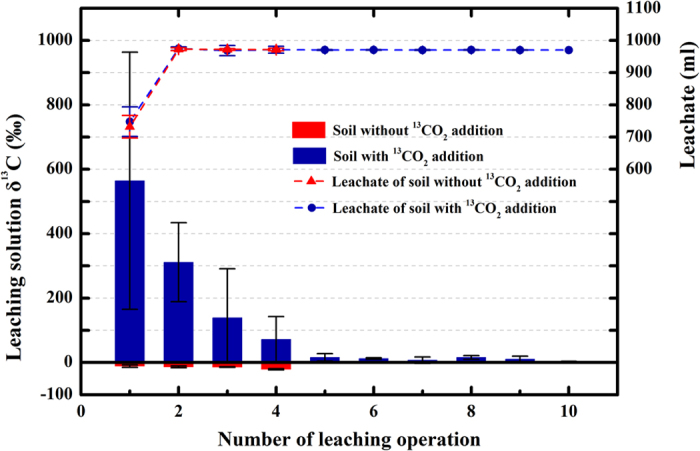
^13^C abundances (δ^13^C; %) of leaching solution of sterilised soil with and without ^13^CO_2_ addition, and the amount (ml) of the corresponding leaching solution. Blue columns and the blue dash line refer to sterilised soil with ^13^CO_2_ addition. Red columns and the red dash line refer to sterilised soil without ^13^CO_2_ addition. Error bars represent standard error of the mean.

The ^13^C abundances and amounts in the leaching solution that removed by frequent leaching are illustrated in Fig. 1. The amount of the collected leaching solution of sterilised soil with ^13^CO_2_ addition did not differ remarkably from that without ^13^CO_2_ addition at each leaching operation, and both approximately equal to 1,000 ml since the second leaching operation. However, the leachate δ^13^C (δ^13^C of DIC in leachate) of sterilised soil with ^13^CO_2_ addition showed intense enrichment (although leachate δ^13^C decreased with the increasing number of leaching operations, all the values of leachate δ^13^C were positive, ranging from 564.48 ± 399.21% to 2.85 ± 1.31%), compared with the leachate δ^13^C of sterilised soil without ^13^CO_2_ addition (all the values of leachate δ^13^C were negative, ranging from −11.40 ± 3.64% to −21.21 ± 1.89%).

### ^13^C abundance (δ^13^C) in the soil gaseous phase with and without ^13^CO_2_ addition

**Figure 2:**
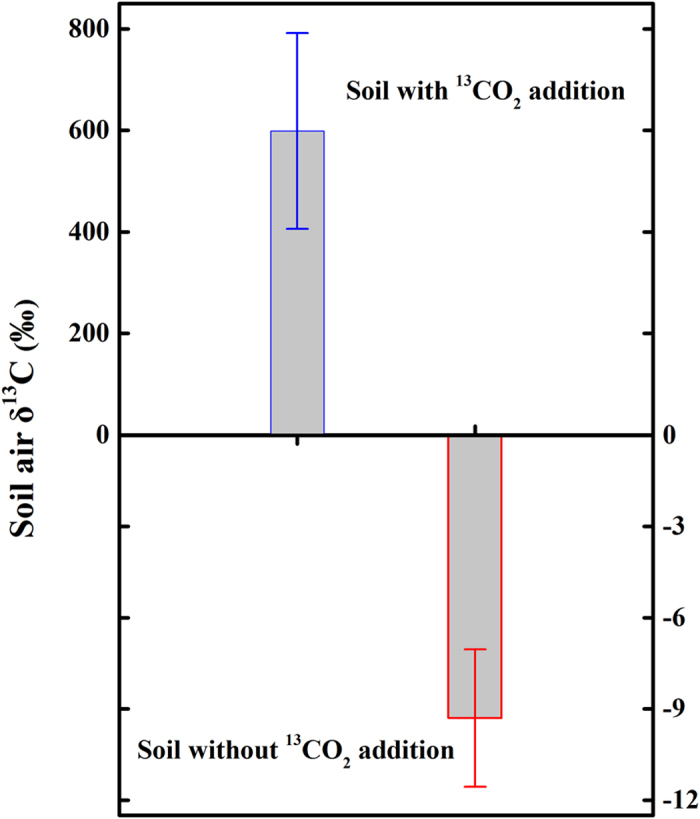
^13^C abundances (δ^13^C; %) of soil air with and without ^13^CO_2_ addition. Grey column with blue border refers to sterilised soil with ^13^CO_2_ addition. Grey column with red border refers to sterilised soil without ^13^CO_2_ addition. Error bars represent standard error of the mean.

The abundances of ^13^C of soil air with and without ^13^CO_2_ addition are showed in Fig. 2. The δ^13^C of soil air without ^13^CO_2_ addition was −9.3 ± 2.26% after soil-atmosphere CO_2_ exchange in the chamber. While the δ^13^C of soil air with ^13^CO_2_ addition was enriched after the CO_2_ exchange in the chamber (599.2 ± 192.87%).

### Absorbed ^13^CO_2_ tracing

The total ^13^C in the soil leaching solution was calculated at about 235.98 μmol, and the ^13^C in the soil air was 0.66 μmol. Thus, the accumulated amount of absorbed ^13^C in the soil liquid phase accounted for 79.45% of the total absorbed ^13^C, while the amount in the soil gaseous phase accounted for only 0.22%. In addition, about 20.33% of the total absorbed ^13^C remained undetected.

## Discussion

79.45% of the labelled carbon (^13^CO_2_) was found in dissolved inorganic carbon (H^13^CO_3_^−^), indicating that the majority of CO_2_ absorbed abiotically by the soil during nighttime is trapped in the soil liquid phase and converted into dissolved inorganic carbon (DIC) at this study site. A recent study also showed that the soil liquid phase in a saline/alkaline desert contains amounts of newly formed DIC, and the formation of DIC may be associated with atmospheric CO_2_^23^. However, in our previous study, it was found that the majority of absorbed ^13^C was fixed in the solid phase of undisturbed soil^19^. The discrepancy between the two studies could be attributed to the influence of biotic processes. Verrecchia and Verrecchia^24^ reported that bacteria and fungi could accelerate the formation of needle fibre calcite (CaCO_3_). The impact of rhizosphere processes may also be important in the formation of carbonate. For instance, the roots absorb soil water and can cause bicarbonate decomposition producing carbonate^25^. In this study, the effect of biotic processes was excluded by soil sterilisation; therefore, the carbon absorbed by the soil could be only trapped abiotically. Moreover, soil water participation and carbon transportation may be another reason for the discrepancy. These may be overlooked in the previous study. Ignoring the interference of biotic processes, conserving in the soil solid phase may be a medium status for the carbon absorbed by soil from atmosphere according to the results in this study. Therefore, this work is an extension of the previous one.

Variations in the ambient temperature can affect in DIC formation and accumulation^26^,^27^,^28^. The high temperature results in DIC decomposition^29^, while the low temperature induces DIC accumulation^12^. In this study, the ambient temperature was low during nighttime. As a result, the process of carbonate dissolution may predominately occur in the soil. In subsoil, the temperature may be persistently lower than topsoil (i.e. the sampled soil in this study)^30^. More DIC may be formed and accumulated without decomposition. Previous studies showed that the residence time of carbon in pedogenic DIC could be over three orders of magnitude higher than that of soil organic matter^31^,^32^,^33^,^34^. Therefore, the newly formed DIC may not easily turn into CaCO_3_ abiotically until the ambience is changed. The accumulation of DIC may induce a continuous consumption of soil CO_2_: inorganic consumption of soil CO_2_ through carbonate dissolution can create a pressure gradient of CO_2_ between the soil and the atmosphere, and then the pressure gradient can induce atmospheric CO_2_ to be pumped into the soil to replenish soil CO_2_ (as the ingredient in carbonate dissolution)^10^.

Although the results showed that the soil could absorb CO_2_ from the atmosphere and then trap large amounts of the atmospheric carbon by forming and accumulating DIC at this study site, we still cannot declare that this is an important soil carbon sink, because we have no direct evidence to support that DIC is sequestrated in the soil at a long-term scale. For the formation of soil carbonate induced by biotic processes, CO_2_, as a by-product, releases back to the atmosphere^34^ and overshadows the abiotic CO_2_ absorption^35^. As a result, carbonate in the soil solid phase may not be the main destination of absorbed atmospheric CO_2_ at the diel or annual scale. The hydrosphere beneath the soil may be a potential outlet of trapped carbon. Figure 1 shows that the vertical migration of soil water occurs easily, and the majority of the absorbed ^13^C in DIC can be washed out by four leaching operations. These results suggested that DIC may finally transport to the aquifer, regardless of being a long-term process. Similarly, Ma *et al.*^18^ and Li *et al.*^23^ reported that soil DIC could be leached into the aquifer by rainfall or snow glacier melt and irrigation water or river water in arid deserts. Moreover, Walvoord *et al.*^36^ found that the accumulated nitrate in the subsoil might also transport into the groundwater because of long-term leaching in xeric ecosystems. In the study site, groundwater is recharged mainly by rainfall^37^ and has almost no hydrological connection to rivers and oceans. If the trapped carbon transports deeply into the aquifer, the low and constant temperature and alkalinity of groundwater may convert the aquifer into a reservoir of carbon beneath the desert soil. However, further studies are required to test whether the newly formed DIC can be leached from the soil to the hydrosphere.

Although this is a case study in the Mu Us Desert, the results can provide the direct and effective support to the hypothesis that carbonate dissolution can induce the abiotic atmospheric CO2 absorption by desert soils. It is undeniable that the amount of carbon trapped in DIC may be overestimated, because the deionized water used for leaching can inevitably dissolve some of the solid fraction (soil carbonate) in theory. To diminish the overestimation, we multiply leached with much less deionized water each time rather than one time leaching with the large amount of deionized water (the duration of each leaching operation is much shorter and the amount of deionized water used for leaching is much less each time). Therefore, the overestimation may be small, and its influence should be slight.

After ^13^CO_2_ addition and exchange in the chambers for 12 h, ^13^C in the soil gaseous phase was enriched (Fig. 2); however, the amount of newly conserved ^13^C in the soil gaseous phase was much less than that fixed by the soil liquid phase (accounted for about 0.28% of the fixed ^13^C in the soil liquid phase). These results were in agreement with those reported in our previous study^19^, suggesting that the soil gaseous phase may only serve as the connecting medium between the atmosphere and the soil liquid phase. CO_2_ in the soil gaseous phase may easily transport upwards to the atmosphere or downwards to the alkaline soil solution.

About 20.33% of the total absorbed ^13^C remained undetected, probably because it was stored in the soil solid phase. Since the leaching operations started at 9:00 A.M., soil temperature might already begin to rise, and a part of DIC probably turned into CaCO_3_ and released as CO_2_. The results from abiotic soil CO_2_ flux measurement showed that soil could absorb atmospheric CO_2_ during nighttime and release CO_2_ during daytime, somewhat supporting our hypothesis. It is notable that even though DIC can turn into CaCO_3_ during daytime, the amount of lapsed DIC may be small, because the abiotic net carbon exchange between the soil and the atmosphere is usually negative at the diel scale (negative net carbon exchange represents carbon absorption by soil)^11^. Therefore, at the diel scale, most of the abiotically absorbed carbon may also be trapped in the soil liquid phase.

## Methods

### Site description

The study site is located on the southwestern fringe of the Mu Us Desert (37°42′N, 107°13′E; 1,509 m above sea level), northern China. It has a temperate continental monsoon climate with a mean annual temperature of 7.6 °C, mean annual solar radiation of 1.4 × 10^5^ J cm^−2^, and mean annual wind speed of 3 m s^−1^ (prevailing northwest wind). The frost-free period lasts around 128 d. The mean annual precipitation is 275 mm (1954–2013), mainly occurring in August and September^35^. The soil type is Aripsamment (derived from aeolian sand; soils with high CaCO_3_ content tend to be salinised). The soil (0–20-cm depth) comprises 94.8% sand, 4.5% silt, and 0.7% clay and has a pH of 8.6^10^. Soil bulk density is 1.54 g cm^−3^ and soil porosity 42%^19^. The study site is sparsely vegetated by *Artemisia ordosica*, *Astragalus mongolicum*, *Salix psammophila*, and *Tamarix chinensis* (canopy coverage ≤ 30%).

### ^13^CO_2_ tracing

**Figure 3:**
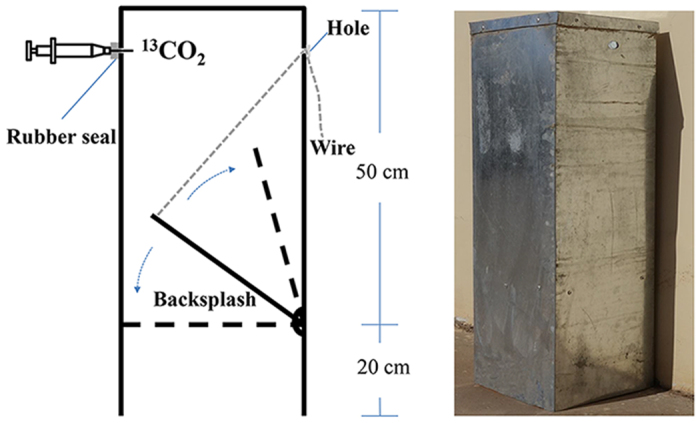
Structure of the ^13^CO_2_ exchange chamber. Neutral glass cement was used to seal the hole immediately after the backsplash was opened or closed during the process of ^13^CO_2_ addition to ensure the chamber sealability.

To test whether carbonate dissolution occurs and detect the location of the abiotically absorbed carbon in the soil, an improved leaching operation (adopted in Li *et al.*^23^; for extraction DIC) and ^13^CO_2_ tracing experiment (used in Liu *et al.*^19^; for carbon tracing) were carried out in September and October 2014 using iron^13^CO_2_ exchange chambers (25 cm in length; 25 cm in width; 70 cm in height; 0.1 cm in thickness; Fig. 3). Three quadrate steel soil collars (25 cm in length; 25 cm in width; 20 cm in height; 0.2 cm in thickness) were placed randomly in the study site with a 2-cm wall to be exposed above the soil surface in order to allow the installation of the iron ^13^CO_2_ exchange chambers. The soil within the collars was equilibrated with its surrounding for 24 h to minimise the disturbing effect. Subsequently, soil samples were collected and sterilised as described by Xie *et al.*^8^. The sterilised soil was placed *in situ* and equilibrated with its surrounding for 17 h to minimise the disturbing effect.

Each ^13^CO_2_ exchange chamber (with backsplash opened) was immersed in NaOH solution (5 mol L^−1^) up to about 2 cm for 2 h to remove ^12^CO_2_. Then, two ^13^CO_2_ exchange chambers (with backlash closed) were installed onto the collars (pushed 15 cm deep into the soil), while the third collar was used as a control. We injected 10 ml ^13^CO_2_ (concentration > 99.99%) into each chamber, opened the backsplash, and allowed the soil to exchange ^13^CO_2_ for 12 h.

The backsplash of each chamber was closed after the ^13^CO_2_ exchange, the gaseous samples (140 ml) in each chamber were collected with an aluminium foil gas-collecting bag, and the ^13^CO_2_ exchange chambers were removed. In order to increase accuracy, a polyvinyl chloride (PVC) sheet (25 cm in length; 25 cm in width; 25 cm in height; 0.1 cm in thickness) was used to divide the sterilised soil into two parts; one for soil CO_2_ extraction (25 cm in length; 5 cm in width; 18 cm in height) and the other for leaching (25 cm in length; 20 cm in width; 18 cm in height). An aluminium foil gas-collecting bag (200 ml) was used to reserve the extracted soil air (140 ml) from the one part of sterilised soil, and a PVC cylinder (15.3 cm outer diameter; 15 cm inner diameter; 25 cm in height) was inserted into the other part of sterilised soil to sample the soil column for leaching. The cylinder was sealed at the top and bottom with base plates and transported to the laboratory.

A layer of gauze (100-mesh) was placed under the bottom plate to prevent mud from leaching. Then, the bottom plate was removed, and the sterilised soil cylinder was leached with 1,000 ml deionised water. The sterilised soil with ^13^CO_2_ addition was leached 10 times (about 10 times of leaching may leach out almost all the ^13^C according to the study of Ma *et al.*^18^), while the control (without ^13^CO_2_ addition) 4 times. We assumed that the abundance of ^13^C would not change considerably, because the natural ^13^C abundance of the soil was low^38^, and thus, the different number of leaching operations could not influence the comparison between the results from sterilised soil with and without ^13^CO_2_ addition. After each leaching operation, we weighed the total volume of leaching solution (*V*_leachate_) and collected leaching solution samples of 30 ml using brown glass bottles. During all these processes, the ambient temperature was below 10 °C.

**Figure 4:**
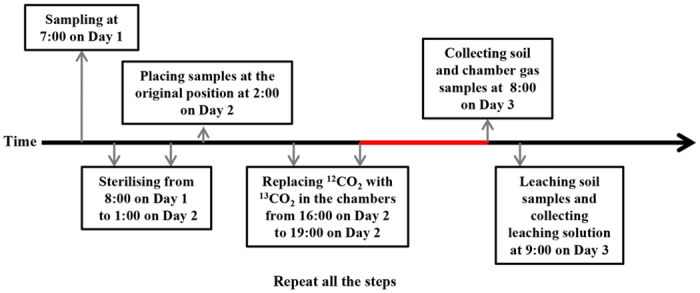
Flow diagram of ^13^CO_2_ tracing. Red line indicates CO_2_ exchange in the chamber.

The number of replications for the sterilised soil with and without ^13^CO_2_ addition was 18 and 9, respectively. The flow of ^13^CO_2_ tracing is shown in Fig. 4. The δ^13^C and CO_2_ concentration of all gaseous samples were measured by a carbon dioxide isotope analyser (CCIA-EP 912-0003; Los Gatos Research, Mountain View, CA, USA) and the δ^13^C of all liquid samples (δ^13^C of DIC in the soil liquid phase) by an isotope mass spectrum analyser (Finnigan MAT253 Gas Bench-IRMS; Thermo Fisher Scientific Inc., Waltham, MA, USA) as described by Liu *et al.*^19^.

### Measurements of soil volumetric water content (VWC) and bicarbonate radical concentration (*C*_DIC_)

VWC was monitored from 20:00 to 7:00 the following day at a depth of 10 cm using the ECH2O system (LI-COR, Lincoln, NE, USA) with five Em50R sensors placed near the soil collars, and data were logged every 1 h. After collecting liquid samples (without ^13^CO_2_ addition), *C*_DIC_ was measured using the conventional method of acid base titration^39^.

### Data processing and analysis

The absorbed ^13^CO_2_ within the soil collar was calculated as follows:


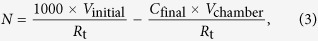


where *N* is the total absorbed ^13^CO_2_ (μmol), *V*_initial_ is the initial volume of ^13^CO_2_ (10 ml), *C*_final_ is the final ^13^CO_2_ concentration (μmol mol^−1^), *V*_chamber_ is the volume of the chamber exposed above the soil surface (L), and *R*_t_ is the molar volume of gas (L mol^−1^).

The ^13^CO_2_ absorbed into the soil vapour phase was calculated as follows:


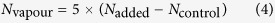



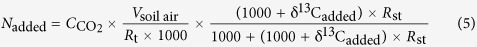



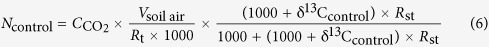



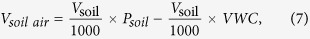


where *N*_vapor_ is the amount of substance of absorbed ^13^C in the soil vapour phase (μmol), 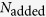 and 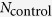 are the amounts of substance of ^13^C in the soil vapour phase with and without ^13^CO_2_ addition, respectively, 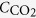 is the concentration of CO_2_ in the soil vapour phase (μmol mol^−1^), *R*_t_ is the molar volume of gas (L mol^−1^), *V*_soil air_ is the volume of soil air (L), δ^13^C_added_ and δ^13^C_control_ are the δ^13^C values of the soil vapour phase with and without ^13^CO_2_ addition, respectively, *R*_st_ is the stable isotope ratio in the reference standard^40^, *V*_soil_ is the volume of soil sample (25 cm × 5 cm × 18 cm), *P*_soil_ is the soil porosity, and *VWC* is the mean value of soil volumetric water content during the measurement period.

The^13^CO_2_ absorbed into the soil liquid phase was calculated as follows:


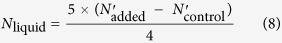



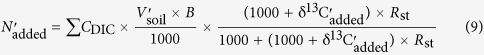



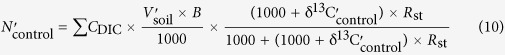


where *N*_liquid_ is the amount of substance of absorbed ^13^C in the soil liquid phase (μmol), 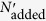 and 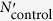 are the amounts of substance of ^13^C in the soil liquid phase with and without ^13^CO_2_ addition, respectively, *C*_DIC_ is the soil bicarbonate radical concentration in the soil liquid phase (μmol kg^−1^), 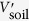 is the volume of soil sample (25 cm × 20 cm × 18 cm), *B* is bulk density (g cm^−3^), and 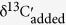 and 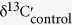 are the δ^13^C values of the soil liquid phase with and without ^13^CO_2_ addition, respectively.

A one-way analysis of variance was used to test the differences in δ^13^C, *C*_DIC_, VWC and *V*_leachate_ between the chambers and samples. All statistical analyses were performed using MATLAB 7.12.0.635 (The Math Works, Natick, MA, USA).

## Additional Information

**How to cite this article**: Fa, K. *et al.* Abiotic carbonate dissolution traps carbon in a semiarid desert. *Sci. Rep.* **6**, 23570; doi: 10.1038/srep23570 (2016).
